# Cancer Metastases to Bone: Concepts, Mechanisms, and Interactions with Bone Osteoblasts

**DOI:** 10.3390/cancers10060182

**Published:** 2018-06-04

**Authors:** Alison B. Shupp, Alexus D. Kolb, Dimpi Mukhopadhyay, Karen M. Bussard

**Affiliations:** Department of Cancer Biology, Thomas Jefferson University, Philadelphia, PA 19107, USA; Alison.Shupp@jefferson.edu (A.B.S.); Alexus.Kolb@jefferson.edu (A.D.K.); Dimpi.Mukhopadhyay@jefferson.edu (D.M.)

**Keywords:** osteoblast, osteoclast, metastasis, breast cancer, prostate cancer, multiple myeloma, bone, dormancy, re-activation, osteomimicry

## Abstract

The skeleton is a unique structure capable of providing support for the body. Bone resorption and deposition are controlled in a tightly regulated balance between osteoblasts and osteoclasts with no net bone gain or loss. However, under conditions of disease, the balance between bone resorption and deposition is upset. Osteoblasts play an important role in bone homeostasis by depositing new bone osteoid into resorption pits. It is becoming increasingly evident that osteoblasts additionally play key roles in cancer cell dissemination to bone and subsequent metastasis. Our laboratory has evidence that when osteoblasts come into contact with disseminated breast cancer cells, the osteoblasts produce factors that initially reduce breast cancer cell proliferation, yet promote cancer cell survival in bone. Other laboratories have demonstrated that osteoblasts both directly and indirectly contribute to dormant cancer cell reactivation in bone. Moreover, we have demonstrated that osteoblasts undergo an inflammatory stress response in late stages of breast cancer, and produce inflammatory cytokines that are maintenance and survival factors for breast cancer cells and osteoclasts. Advances in understanding interactions between osteoblasts, osteoclasts, and bone metastatic cancer cells will aid in controlling and ultimately preventing cancer cell metastasis to bone.

## 1. Introduction

Bone is a unique organ of the body capable of providing structural support, regulation of calcium levels in blood, as well as protection of internal soft tissue organs [1]. To accomplish these functions, bone undergoes a dynamic process of remodeling to respond to mechanical strain and stress. Importantly, bone is predominantly composed of type I collagen, followed by other non-collagenous proteins and proteoglycans which harden through a process called mineralization [1]. Mineralization increases bone strength and resistance to compression [2].

There are two types of bone found within the body: cortical bone and trabecular bone [1]. Cortical bone is a dense layer of outer tissue that plays a vital role in supporting the weight load of the body, as well as provides protective functions. Cortical bone is composed of densely packed collagen fibrils that are aligned along the longitudinal axis of bone, and parallel to hydroxyapatite crystals in a manner that permits bone to resist tensile strain [3,4]. On the other hand, trabecular bone consists of a large, porous matrix located on the inner surface of bone ends. Long bones, including the humerus and femur, as well as flat and irregular bones, such as vertebrae and the sternum, are predominantly composed of trabecular bone [5]. While both cortical and trabecular bone are metabolically active, trabecular bone undergoes remodeling at a rate higher than cortical bone [1].

### 1.1. Gross Anatomy of Bone

Bone is a complex tissue and has many unique properties that make it a hospitable microenvironment for metastatic cancer cells. Both breast and prostate cancer are known for their inclination to metastasize to bone [6,7,8]. The skeleton is comprised of four types of bones: short bones, long bones, flat bones and irregular bones [9]. Short bones are similar in shape to a cube, and can be found in the wrist or ankle. Flat bones are flattened plates of bone that are often curved in shape, such as the skull and mandible, while irregular bones have a non-uniform shape (e.g., vertebrae and sacrum). Lastly, long bones, such as the femur and humerus, support the limbs of the body. These types of bones differ in both their function and structural organization. All bones contain a dense outer layer of bone, called cortical bone, but they differ in their relative amounts of cortical bone and trabecular bone. Cortical bone, also called compact bone, is formed from densely packed collagen fibrils, in particular type I collagen [1,10]. Located in the interior of bone and near bone ends, is trabecular bone, also referred to as cancellous bone or spongy bone. This type of bone is less dense than cortical bone, and consists of a porous matrix. While cortical bone imparts strength and structure to the skeleton that is necessary to support the body, trabecular bone provides flexibility needed for withstanding mechanical force [11].

**Figure 1:**
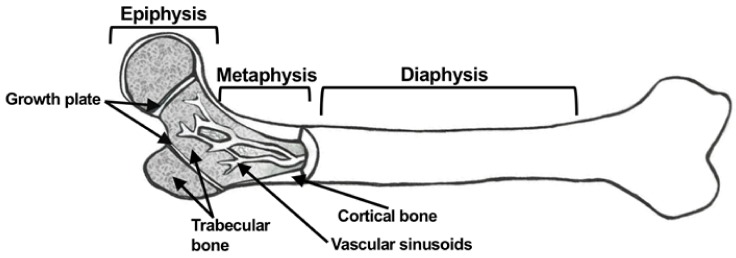
Anatomy of long bones. Depicted are the three regions of long bones: epiphysis, metaphysis, and diaphysis. The outside of the bone is composed of dense cortical bone, while trabecular bone can be found in the interior and near bone ends. Also indicated are the growth plates, at the ends of the bone, and the sinusoidal vasculature that is found in the epiphysis.

Long bones consist of three regions: the epiphysis, metaphysis, and diaphysis (Figure 1). The epiphysis is located at the ends of long bones, above the growth plate, where bone growth and elongation occurs. The diaphysis is the shaft of long bones, which consists of cortical bone that surrounds a bone marrow-filled cavity [12]. The metaphysis is adjacent to the epiphysis, but below the growth plate, and is composed of trabecular bone. The metaphysis has sinusoidal vasculature that results in slow-moving blood flow through blood vessels compared to capillary networks. This sluggish blood flow in vascular sinusoids is ideal for movement of hematopoietic and lymphoid cells in and out of the bone, but it is also exploited by cancer cells trafficking to bone [6,13].

### 1.2. Bone Physiology, Remodeling, and Metabolism

Bone is an extremely metabolically active tissue that is constantly being remodeled. There are three main cell types that facilitate bone remodeling: osteoblasts, osteoclasts, and osteocytes. Osteoblasts account for 4–6% of the total cells in bone [14]. Osteoblasts synthesize and mineralize new bone matrix, called osteoid, while osteoclasts degrade existing bone. Bone matrix is largely composed of type I collagen, but also contains proteoglycans and other extracellular matrix proteins [10]. Osteoblasts, derived from mesenchymal stem cells, aid in matrix mineralization through the formation of hydroxyapatite crystals. As osteoid is synthesized, some osteoblasts become embedded into the bone matrix and develop into osteocytes. Osteocytes form interconnected networks through small tunnels called canaliculi. Osteocytes, which account for 90–95% of all cells in the bone, are important for detection of mechanical forces, and communicating to osteoblasts and osteoclasts to induce bone deposition or bone resorption as a response [15].

Osteoclasts, the cells responsible for resorbing existing bone, are derived from monocytes present in bone marrow. Osteoclasts account for 1–4% of the total cells in bone [14,16]. Mature osteoclasts are formed via RANK-L (receptor activator for nuclear factor kappa beta (NF-κB) ligand) signaling. RANK-L, which is abundantly produced by osteoblasts, binds the RANK receptor on monocytes [17]. Another stimulus that induces mature osteoclast formation is the presence of macrophage colony stimulating factor (M-CSF). In the presence of M-CSF and RANK-L, multiple monocytes fuse together to form a mature and multinucleated osteoclast [17]. Osteoclasts have a unique morphology that optimizes them for bone resorption. Their cell membrane forms a ruffled border with the surface of the bone matrix. The ruffled structure increases membrane surface area through which bone-degrading enzymes, such as tartrate resistant acid phosphatase (TRAP) can be secreted [18]. As osteoclasts resorb bone, various growth factors (e.g., bone morphogenic proteins (BMPs), tumor-growth factor-beta (TGF-B), insulin growth factor (IGF)), cytokines, and chemokines that are embedded in the bone matrix are released into the microenvironment [19].

### 1.3. Osteoblast Differentiation

Bone contains pre-osteoblast cells that migrate to the site of bone resorption, differentiate into mature osteoblasts, and secrete bone matrix to fill in the resorption cavities [1]. Osteoblasts are derived from precursor mesenchymal stromal cells which can be found in the bone marrow stroma [1]. Upon stimulation by local growth factors and bone morphogenetic proteins, mesenchymal stromal cells proliferate to form pre-osteoblast cells, which then later differentiate into mature osteoblasts [20]. Mature osteoblasts are responsible for laying down new bone, which is composed of both type I collagen and non-collagenous proteins (~22%) [1]. The remaining ~80% composition is made of up water (~8% by weight) and hydroxyapatite (~70%) [3].

**Figure 2:**
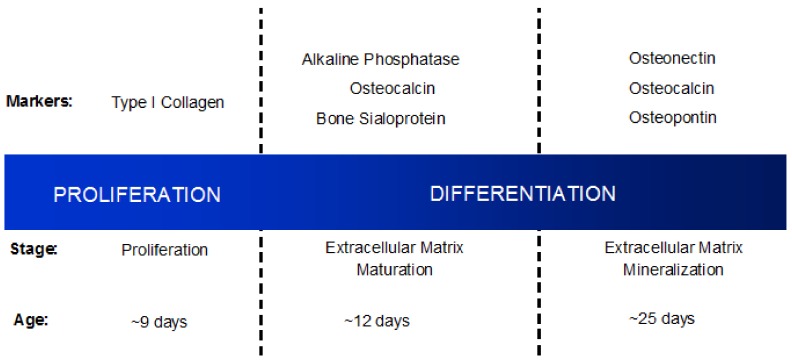
Osteoblast differentiation. Murine osteoblast differentiation is characterized by three stages of growth marked by specific factor expression. During osteoblast proliferation (up to approximately 9 days), osteoblasts produce type I collagen. Osteoblasts enter early differentiation, or extracellular matrix maturation, at approximately 12 days of age, and express the proteins alkaline phosphatase, osteocalcin, and bone sialoprotein. Extracellular matrix mineralization occurs when osteoblasts are approximately 25 days old, where osteoblasts express the proteins osteonectin, osteocalcin, and osteopontin.

Murine osteoblast differentiation is characterized by three principle periods distinguished by markers indicative of each stage: proliferation (~9 days), extracellular matrix maturation (~12 days), and extracellular matrix mineralization (~25 days) [21] (Figure 2).

Proliferation is characterized by the secretion of type I collagen, while alkaline phosphatase, bone sialoprotein, and osteocalcin indicate extracellular matrix maturation [21]. The presence of osteopontin, osteocalcin, and osteonectin signal extracellular matrix mineralization, which is also marked by bone nodule formation [21]. Osteoblast differentiation is continuously ongoing, working in concert with osteoclast bone-resorption.

### 1.4. Osteoblasts Work in Concert with Osteoclasts to Regulate Bone Remodeling

Osteoclasts are responsible for bone resorption. These resorptive cells are derived from monocytes located in the bone marrow stroma [17]. In order to activate osteoclasts, osteoblasts express RANK-L on their plasma membrane surface. This ligand binds to the receptor RANK found on the surface of osteoclast progenitor cells [22]. Osteoprotegerin (OPG) is a decoy receptor for RANK-L, and is also produced by osteoblasts. The ratio of osteoblast-derived RANK-L: OPG is one manner in which osteoblasts regulate osteoclastogenesis [22]. When RANK-L expression is high and OPG expression is low, osteoclastogenesis will predominate. When OPG levels are raised, OPG will bind RANK-L, thus suppressing osteoclast activation [22]. In addition to RANK-L and OPG, osteoblasts secrete M-CSF, which binds to the colony-stimulating factor-1 receptor (c-fms) on osteoclasts to further stimulate osteoclastogenesis [23,24]. Mature osteoclasts form from the fusion of several monocytes to form one large, multinucleated osteoclast [17]. Upon the release of signals to stimulate bone resorption, multi-nucleated osteoclasts become activated, and bind to the bone matrix. Bone resorption occurs via osteoclast secretion of metalloproteinases (MMPs), cysteine proteinases (including cathepsin K), phosphatases, lysozymal enzymes, and low pH molecules including citric acid and hydrochloric acid [17,25,26,27].

Under conditions of normal bone homeostasis, there is a tightly regulated balance between bone deposition and resorption, where there is no net bone gain or loss. However, this balance is upset in several pathological conditions, including osteomyelitis, osteoarthritis, and bone metastatic cancers [28,29,30,31]. In each of these conditions, and especially in osteolytic disease, osteoclasts are overstimulated to degrade bone. Osteoblasts do not deposit new bone; thus resulting in net bone loss [32].

## 2. Osteoblasts in the Bone Microenvironment as Contributors to Bone Disease and Degradation

As previously discussed, bone homeostasis is controlled by a tightly regulated balance between bone deposition and bone resorption [6]. However, this balance can be upset in situations of infection [33], chronic inflammation [34,35], or cancer [6]. In particular, osteoblast activity has been shown to be dysregulated in pathological conditions of bone infection such as osteomyelitis. Osteomyelitis is a severe bone infection, typically caused by the bacterium *Staphylococcus aureus*, which, if left untreated, can lead to patient death [36,37]. In adults, *S. aureus* infection and subsequent osteomyelitis are typically associated with surgical osseointegration implants (e.g., femoral implants [artificial hip] or dental implants) [38,39,40]. Interestingly, *S. aureus* is highly adapted to specifically interact with bone osteoblasts as a result of microbial surface components recognizing adhesive matrix molecules, namely bone sialoprotein, osteopontin, type I collagen, fibronectin, and integrin alpha 5 beta 1 [29,41]. All of these factors are strongly expressed by bone osteoblasts as compared to other cells of the bone niche [6]. Crosstalk between *S. aureus* and osteoblasts through these mechanisms permits the internalization of *S. aureus* by osteoblasts, as well as allows *S. aureus* to escape immune detection and cause sustained bone infection [42]. Upon internalization of *S. aureus*, osteoblasts increase their production of inflammatory cytokines including interleukin-6 (IL-6), monocyte chemoattractant protein-1 (MCP-1), regulated on activation normal T cell expressed and secreted (RANTES), and macrophage inflammatory protein-1 alpha (MIP-1 alpha); factors that stimulate osteoclastogenesis including granulocyte-colony stimulating factor (G-CSF), RANK-L, and granulocyte macrophage-colony stimulating factor (GM-CSF); and cytokines that recruit both innate and adaptive immune cells including interleukin-8 (IL-8), interleukin-12 (IL-12), and interferon gamma-induced protein 10 (IP-10) [43,44,45,46,47,48,49]. Interestingly, these same cytokines have also been shown to be upregulated by osteoblasts during bone invasion by metastatic breast cancer cells [50,51,52]. In addition, sustained chronic infection of osteoblasts by *S. aureus* has been found to lead to a reduction in osteoblast proliferation; decreased differentiation as evidenced by reduced expression of the bone turnover markers alkaline phosphatase, osteocalcin, osteonectin, and osteopontin; and a reduction in calcium deposition and osteoblast mineralization [49,53,54]. Ultimately, osteoblasts die due to the sustained infection [55]. Furthermore, the increase in cytokines produced by osteoblasts as a result of sustained infection are capable of eliciting increased osteoclastogenesis [49]. As a result of increased osteoclast formation, yet decreased osteoblast activity, bone is resorbed at a rate higher than it is deposited, leading to sustained bone degradation and perpetuated bone loss [56]. A detailed review of interactions between osteoblasts and *S. aureus* can be found in [29].

In addition to osteomyelitis, osteoarthritis is a common joint disease typically characterized by chronic inflammation and altered osteoblast function. It has been demonstrated that osteoblasts produce increased amounts of the inflammatory cytokines IL-6, IL-8, prostaglandin E2 (PGE2), and vascular endothelial growth factor (VEGF); extracellular matrix markers matrix metalloproteinase-9 (MMP-9) and type I collagen; as well as tumor-growth factor beta-1 (TGF-beta 1) in regions of sclerotic bone as compared to normal bone [30,31,57]. And, similar to osteomyelitis, regions of osteoarthritis are marked by an imbalance in alkaline phosphatase expression, and a reduction in osteoblast mineralization and bone sialoprotein expression [58,59]. Moreover, there is an imbalance in the ratio of RANK-L/OPG produced by osteoblasts leading to alterations in bone remodeling [60]. Thus, osteoblast function, including production of cytokines, growth factors, and osteoclastogenesis-initiating factors, as well as osteoblast differentiation and mineralization, is altered in chronic states of disease in bone.

## 3. Bone Is a Favored Site for Cancer Cell Metastasis

**Figure 3:**
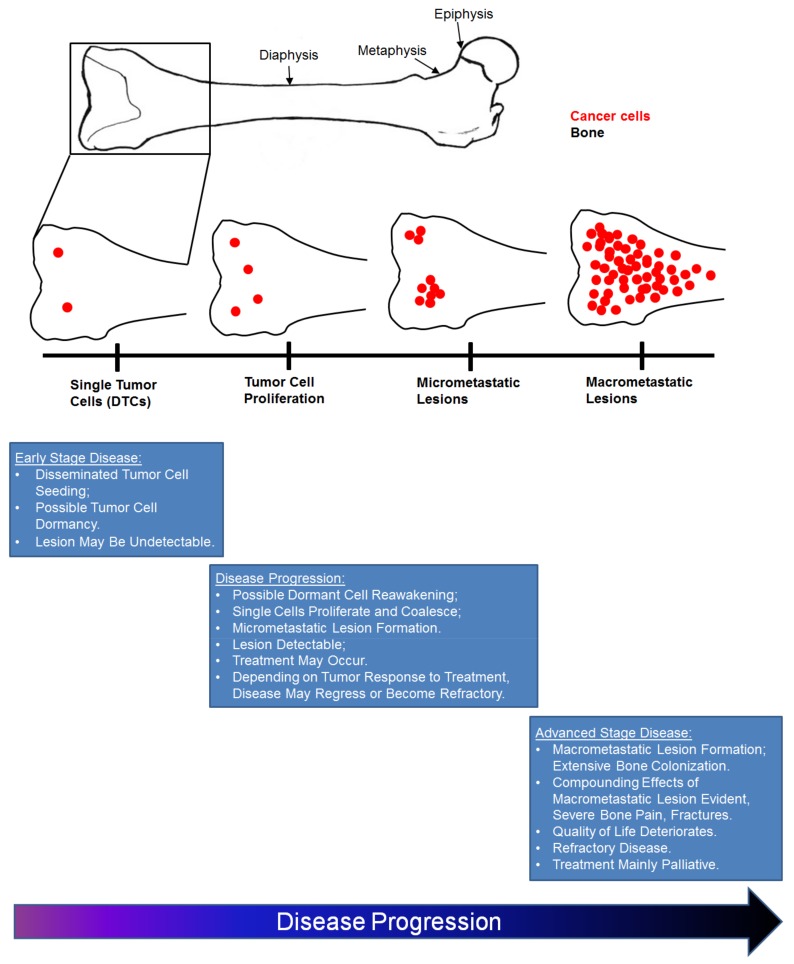
Stages of disease progression during bone metastatic cancer. Bone metastatic cancers may be broadly defined by three overarching stages: early disease, disease progression, and advanced or late stage disease. During early stage disease, disseminated tumor cells circulating in the vasculature enter bone and seed mainly as single cells. Cells may be undetectable by current technological methods due to their solitary nature. Cancer cell dormancy may also occur. As bone metastatic disease progresses over a period of months to years to potentially decades, dormant cancer cells become re-awakened leading to proliferation and coalescing of smaller, micrometastatic lesions. Lesions may become detectable by current technological methods. Treatments to reduce tumor size and alter bone remodeling may occur. Over time, tumors may become refractory to treatment modalities, leading to sustained tumor cell proliferation and excessive tumor burden. Macrometastatic lesions form. Patients may experience effects of increased tumor burden including severe bone pain, fractures, and hypercalcemia. Patient quality of life progressively deteriorates. Treatment modalities during advanced stage disease are mainly palliative to reduce complications from excessive bone tumor burden.

In 1889, in an attempt to explain directional tropism of disseminated breast cancer cells for certain organs of the body as opposed to others, Stephen Paget made the statement “When a plant goes to seed, its seeds are carried in all directions; but they can only live and grow if they fall on congenial soil [61].” Nearly 130 years later, Paget’s “seed and soil” hypothesis best describes the crosstalk between the tumor cell (the “seed”) and secondary microenvironments (the “soil”). Bone is an especially congenial soil for cancer cell metastasis mainly due to it being a rich source of growth factors, neovascularization factors, cytokines, and chemokines that facilitate cancer cell colonization, growth, and sustained survival [6]. Furthermore, mounting evidence has implicated the cells of the bone responsible for remodeling, the osteoblasts and osteoclasts, as key players in bone metastatic cancer cell progression, including cancer cell homing to and seeding in bone, dormancy, cancer cell re-activation, and contribution to macrometastatic lesion growth. These topics will be discussed in detail in future sections, but can be broadly defined as events that occur either in early stage disease, disease progression, or late or advanced stage disease (Figure 3). Late or advanced stage bone metastases are typically characterized by macrometastatic lesion formation and extensive tumor cell colonization of bone [62]. Patients presenting with advanced stage disease frequently experience bone pain, hypercalcemia, and fractures [63]. As a result, patient quality of life is impacted. Treatment modalities are mainly palliative to alleviate complications associated with increased skeletal tumor burden [63] (Figure 3). These characteristics are in contrast to early stage cancer-induced bone disease characterized on the basis of the seeding of solitary or single disseminated tumor cells in the bone [64]. As a result of their small size, tumor cells at this stage may be undetectable by currently available technologies. Cancer cell dormancy may also occur [64] (Figure 3). We previously conducted a comprehensive detailed analysis of the kinetics of breast cancer cell trafficking into the bone and changes in the dynamic progression of disease over time with respect to cancer cells, osteoblasts, and osteoclasts, which can be found in [62]. Furthermore, Mastro and colleagues conducted a study that describes alterations in disease progression, especially in late stage bone metastatic breast cancer, with respect to spatial distribution of cancer cells and osteoblasts, which can be found in [28].

Similar studies were carried out focusing on early events in bone metastatic prostate cancer by Wang et al. yielding comparable results with respect to preferential localization of cancer cells to endosteal regions of bone, including preferred trafficking of cancer cells to the endosteal marrow as opposed to central marrow [65].

Cancer cells, including those of prostate [66], breast [67], and multiple myeloma [68] frequently metastasize to bone. Other cancers, including lung [69,70], liver [71], kidney [72], and thyroid [73] also metastasize to bone, but at a less frequent rate than prostate, breast, or multiple myeloma.

### 3.1. Breast Cancer Metastases to Bone

It has been estimated that between 20–30% of breast cancer patients will develop metastases [74], with bone metastases resulting in approximately 15% of these patients [75]. Among these, approximately 50% of metastases will involve bone as a primary metastatic site, whereas 80% involve bone as a secondary and/or recurring metastatic site [7,76,77,78]. Once breast cancer metastasizes to the bone, the 5 years relative survival rate falls to under 10% [76]. At the present time, there is no cure for breast cancer that has metastasized to the bone.

Metastatic lesions that form as part of bone metastatic breast cancer disease are predominantly osteolytic [62,79], although there have been some reports of lesions that are mixed blastic and lytic [80]. Interestingly, in a mouse model of breast cancer metastases to bone, Yi et al. found that inoculated MDA-MB-231 human triple negative breast cancer cells overexpressed platelet-derived growth factor-BB (PDGF-BB) causing partial osteosclerosis, or abnormal bone hardening, in mice [81]. In addition, breast cancer cells have also been found to produce endothelin-1 (ET-1), which activates osteoblast differentiation via suppression of dickkopf-1 (DKK-1) and increased wingless-related integration site (Wnt) signaling [82]. Exact mechanisms that elicit blastic lesions in bone metastatic breast cancer are not yet fully understood.

The vast majority of bone lesions that form as a result of metastatic breast cancer are lytic. As part of the well-described ‘vicious cycle’ of breast cancer metastases to bone, metastatic breast cancer cells produce parathyroid hormone related protein (PTHrP), which stimulates osteoblasts to produce increased amounts of RANK-L. RANK-L on the surface of osteoblasts binds to the receptor RANK on osteoclast precursors, stimulating osteoclastogenesis and increased bone resorption. Growth factors stored in bone are released, including insulin growth factor-1 (IGF-1) and TGF-beta, which are used by cancer cells to produce additional PTHrP (Figure 2) [83,84]. Bone resorption occurs at a rate greater than bone deposition. In fact, as part of a comprehensive analysis to determine the trafficking patterns of metastatic breast cancer cells to bone, Phadke et al. determined that osteoblasts located adjacent to metastatic breast cancer cells undergo apoptosis, resulting in the number of osteoblasts per bone surface being statistically decreased in late stage advanced disease with the presence of macrometastatic lesions. This finding correlated with a decrease in bone volume, but, interestingly, it was found that the number of TRAP+ osteoclasts also decreased upon formation of macrometastatic tumors [62]. These results suggest that in addition to diminished numbers of osteoblasts in late stages of bone metastatic breast cancer, the number of osteoclasts also decreases with increased tumor burden.

Clinically, patients with bone metastatic breast cancer and other lytic metastases are treated with drugs aimed at blocking the activity of osteoclasts, thereby slowing bone resorption. Bisphosphonate therapies such as denosumab [85], ibandronate [63,85,86], and zoledronic acid [87] alter osteoclast formation through either suppression of osteoblast-derived RANK-L, or increased expression of OPG, a decoy receptor for RANK-L. RANK-L binds to RANK receptor on immature osteoclasts to initiate osteoclastogenesis [88]. When present in the bone niche, RANK-L will bind to OPG instead of RANK receptor on immature osteoclasts [89]. In this way, increased expression of OPG leads to reduced osteoclastogenesis and bone degradation via alteration of the RANK-L/OPG ratio [89,90]. While these treatments slow the formation of new lesions, they are not curative for lesions already present [91,92]. Currently, there are no drugs available to stimulate osteoblast activity and bone deposition. Bisphosphonate therapies also do not prevent recurring metastases from forming. Bone pain, fractures, spinal cord compression, and hypercalcemia may result [84,88,93].

### 3.2. Multiple Myeloma Colonization of the Skeleton

Multiple myeloma frequently metastasizes to bone [94]. Of patients presenting with multiple myeloma, approximately 70% of them have bone metastases upon diagnosis [95]. During the course of disease progression, over 90% of patients with multiple myeloma will develop skeletal lesions that are primarily osteolytic in nature [95]. During bone metastatic multiple myeloma, bone is resorbed at a rate faster than it is deposited resulting in increased formation of osteolytic lesions [95]. It has been demonstrated that metastatic multiple myeloma cells cause suppressed differentiation of osteoblasts from bone marrow mesenchymal stroma cells, leading to decreased bone deposition. Studies by the Roodman laboratory and others have demonstrated that multiple-myeloma-derived factors including interleukin-3 (IL-3) [96,97], sclerostin [98], TGF-beta [99,100], interleukin-7 (IL-7) [101], tumor necrosis factor-alpha (TNF-alpha) [101], DKK1 [102], and the zinc finger protein GFI1 [101] all contribute to suppression of osteoblast differentiation and bone deposition. Importantly, it was determined that bone metastatic multiple myeloma cells induce epigenetic changes at the *RUNX2* locus which prevent osteoblasts from differentiating. Reduced expression of *RUNX2* correlates with increased expression of GFI1 in osteoblast precursors [101]. Ectopic expression of GFI1 in osteoblasts leads to GFI1 binding to *RUNX2*, recruitment of the histone modifiers EZH2 and HDAC1, and reduced expression of *RUNX2* in osteoblasts [103]. Knockdown of *GFI1* in multiple myeloma cells was sufficient to reverse the recruitment of EZH2 and HDAC1, increase *RUNX2* expression, and rescue osteoblast differentiation [103].

Clinically, therapeutic treatments for skeletal lesions as part of multiple myeloma bone disease have little impact on osteoblast function and thus bone repair, although life expectancy has been significantly improved [104]. Patient data suggests that osteolytic lesions rarely heal due to suppressed osteoblast differentiation even in patients with long-term complete remission of multiple myeloma bone disease [105]. Thus, there is a critical need for the development of therapeutic drugs that both target tumor progression as well as improve bone deposition via specific targeting of bone osteoblasts. As a start to this endeavor, Delgado-Calle et al. recently reported that use of genetic deletion of sclerostin, a potent Wnt/beta-catenin antagonist, prevents the loss of bone in a mouse model of multiple-myeloma-induced bone disease. Additionally, mouse treatment with an antibody to sclerostin led to reduced osteolytic lesion formation and increased bone volume in mice with multiple myeloma bone tumors [98]. Specifically, tumor-bearing mice deleted for sclerostin exhibited a 60% improvement in the number of osteolytic lesions and a 74% decrease in the area of osteolytic lesions compared to tumor-bearing wild-type mice. Furthermore, tumor-bearing mice deleted for sclerostin exhibited a 50% increase in trabecular bone volume compared to tumor-bearing wild-type mice [98]. Most importantly, tumor-bearing mice deleted for sclerostin exhibited an increase in the number of osteoblasts, which correlated with an increase in bone formation when compared to tumor-bearing mice present for the gene. Osteoblast activity in tumor-bearing mice deleted for sclerostin remained at levels comparable to control mice injected with saline indicating rescue of osteoblast function in part by deletion of sclerostin [98]. Similarly, use of an antibody to sclerostin in tumor-bearing mice resulted in an increase in the number of osteoblasts/bone surface which correlated with increased trabecular bone volume [98]. These results suggest that inhibition of sclerostin may be a promising tool to promote bone deposition via increased osteoblast activity in multiple myeloma bone disease.

DKK1 is another inhibitor of osteoblast function that has been evaluated in recent clinical trials [106,107]. DKK1 has been found to inhibit the production of osteoblasts via preventing the binding of LRP5/6 to Wnt, thereby leading to a decrease in the expression of runt-related transcription factor-2 (RUNX2) in osteoblast precursors [108]. RUNX2 is a master regulator of osteoblast differentiation and function [109]. As part of a phase I/II clinical trial, an antibody to DKK1 was found to increase osteoblast differentiation in-vitro upon co-culture with multiple myeloma cells. In addition, there as a statistically significant increase in calcium deposition upon treatment with DKK1 antibody in co-cultures of pre-osteoblasts plus multiple myeloma cells [107]. These results suggest an increase in bone deposition. When used in a humanized mouse model with multiple myeloma-induced bone disease, a significant increase in the number of osteoblasts was observed as compared to untreated mice [107]. In addition, treatment with DKK1 antibody increased trabecular bone volume in tumor-bearing mice when compared to mice treated with placebo. Furthermore, treatment with the DKK1 antibody led to increased expression of Wnt/beta-catenin signaling, which is crucial for osteoblast differentiation, when compared to untreated mice [107]. Taken together these results suggest that inhibitors to DKK1 may be promising targeted therapeutics that promote osteoblast differentiation and subsequent bone deposition in patients with established multiple myeloma bone disease.

### 3.3. Prostate Cancer Metastases to Bone

Prostate cancer is another cancer type that frequently metastasizes to the bone. However, when compared to bone metastatic breast cancer and multiple myeloma-induced bone disease, lesions that result from bone metastatic prostate cancer can be either blastic, lytic, or mixed [110,111], though the majority of patients with bone metastatic prostate cancer present with osteoblastic lesions [112]. Blastic skeletal lesions that occur as a result of bone metastatic prostate cancer are named such due to their characteristic osteosclerotic, or hardened bone appearance on X-rays or CT scans [113]. When compared to patients with primarily lytic diseases, osteoblast production of markers such as alkaline phosphatase and procollagen C-propeptide are elevated in patients with bone metastatic prostate cancer. This is because prostate cancer cells preferentially home to osteoblast-rich regions of the bone, whereby direct contact between metastatic prostate cancer cells and osteoblasts leads to an increase in both osteoblast and prostate cancer cell proliferation [65,113]. Interestingly, studies by Sekita et al. determined that even though osteoblast proliferation was increased in bone metastatic prostate cancer, formation of an organized bone matrix was disrupted due to malalignment of osteoblasts along a collagen matrix. This led to production of bone with a spongeous structure as opposed to a compact lamellar structure. Disrupted bone matrix resulted in reduced bone toughness and impaired overall bone function [114,115].

Similar to the ‘vicious cycle’ of osteolytic bone metastases, bone metastases in prostate cancer enter into an autocatalytic cycle promoting both osteoblast proliferation and differentiation as well as metastatic prostate cancer cell proliferation. Bone metastatic prostate cancer cells secrete factors that promote osteoblast differentiation, including bone morphogenic proteins (BMPs), TGF-beta, IGF-1, platelet-derived growth factor, endothelin-1, and VEGF [8,93,116,117]. In turn, osteoblasts deposit new bone and matrix degradation is suppressed [83,118]. Activated osteoblasts additionally produce factors that stimulate prostate cancer cell proliferation including, VEGF, MCP-1, IL-6, and IL-8 (murine MIP-2) [119]. Osteolytic factors, including PTHrP additionally stimulate osteoblast differentiation and proliferation [120]. Furthermore, secretion of endothelin-1 by prostate cancer cells has been demonstrated to suppress DKK1, stimulating Wnt signaling and osteoblast bone deposition [121]. Thus, sustained bone formation occurs even in the presence of tumor growth. In addition to factors that promote osteoblast proliferation and differentiation, bone metastatic prostate cancer cells additionally express factors that stimulate osteoclastogenesis, including increased RANK-L and M-CSF. Activated osteoclasts resorb bone releasing growth factors including TGF-beta and IGF-1 which further stimulate prostate cancer growth [122].

Several agents are currently in clinical trials to combat blastic lesions produced during bone metastatic prostate cancer. These agents seek to reduce osteoblast bone deposition. Among them is atrasentan, an antagonist to the endothelin receptor A that seeks to reduce osteoblast differentiation and proliferation through blockade of endothelin receptor signaling and reduced Wnt signaling [121,123]. In a phase I/II clinical trial, use of the endothelin receptor A antagonist led to a reduction in bone pain and bone turnover, as well as slowed prostate tumor growth [124]. In phase II trials, patient treatment with atrasentan resulted in a significantly prolonged median time to disease progression when compared to placebo group [125]. These promising results led to a phase III trial which compared the use of atrasentan plus or minus docetaxel in patients with metastatic prostate cancer. Results of the phase III trial were unfortunately disappointing, whereby essentially no change was seen in median progression-free survival time or overall survival in patients treated with atrasentan plus docetaxel versus patients treated with docetaxel alone [126]. These results were similar to an earlier phase III clinical trial which sought to determine the efficacy of atrasentan in patients with metastatic hormone refractory prostate cancer [123]. In addition to atrasentan, radium-223 has been investigated as a treatment for patients with bone metastatic prostate cancer. Radium-223 is an alpha particle emitter that accumulates preferentially at sites with increased osteoblast activity and bone deposition [127]. In a mouse model of bone metastatic prostate cancer, tumor-bearing mice treated with radium-223 had reduced osteoblastic bone growth. In addition, osteoblasts found within tumor lesions exhibited double-stranded DNA breaks, suggesting disruption of new bone formation within tumor lesions. Moreover, use of radium-223 preserved bone volume and bone architecture [127]. Ongoing and future clinical trials will help to determine the optimal treatment modalities for patients with bone metastatic prostate cancer.

## 4. Osteoblasts as Mediators in Cancer Cell Dormancy in Bone

**Figure 4:**
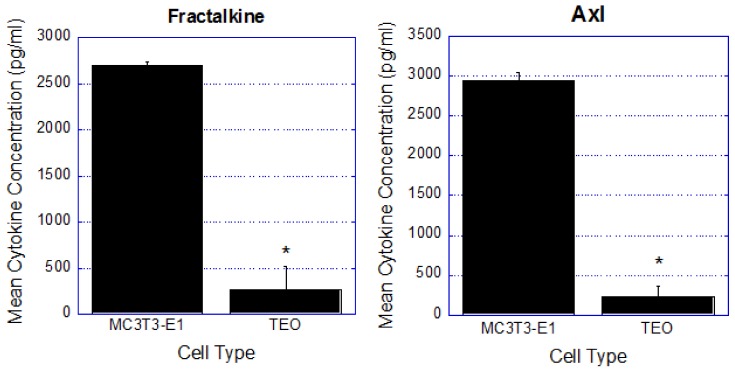
Tumor-educated osteoblast cells have altered cytokine production when compared to untreated osteoblasts. Conditioned media was prepared from untreated MC3T3-E1 osteoblasts or TEO cells and subjected to a RayBiotech Quantibody^®^ Quantitative Multiplex ELISA Array. Three separate batches of conditioned media were assayed per condition. Shown are the mean protein concentration of Fractalkine and Axl produced by either untreated MC3T3-E1 osteoblasts or TEO cells. * *p* < 0.05.

It is becoming increasingly evident that osteoblasts in the bone microenvironment play vital roles in cancer cell attraction [128,129], maintenance [50], and survival [6,130,131] during cancer progression in bone. In particular, our laboratory previously demonstrated that osteoblasts are directed by bone metastatic breast cancer cells to increase osteoblast production of inflammatory cytokines implicated in cancer cell maintenance and survival [50]. Given that osteoblasts are directed by metastatic breast cancer cells in late stage disease to facilitate cancer cell survival in bone, we postulated that osteoblasts may also be involved in mediating events that occur during initial breast cancer cell bone dissemination. Our laboratory has evidence to suggest that crosstalk between osteoblasts and disseminated breast cancer cells elicits osteoblast production of factors that regulate breast cancer cell proliferation and survival in bone. Following extended exposure to breast cancer cells or their conditioned media, our laboratory demonstrated that exposed osteoblasts exhibit characteristics different from unexposed osteoblasts, including altered expression of cytokines and growth factors, including the proteins fractalkine and Axl (Figure 4), among others [132]. We call these exposed osteoblasts ‘tumor-educated osteoblasts’ (TEO).

Compared to unexposed osteoblasts, TEO cells produce decreased amounts of both fractalkine (11 fold decrease) and Axl (12 fold decrease) (Figure 4). Fractalkine produced by osteoblasts is known to be a chemoattractant for prostate cancer cells, as well as support prostate cancer cell migration and survival [133]. Fractalkine also plays a role in the dissemination of breast cancer cells to bone; and inhibiting the fractalkine receptor, CX3CR1, has been shown to reduce breast cancer cell bone dissemination and development of bone metastatic lesions [134,135]. Additionally, Axl is a receptor tyrosine kinase that has been described as a key player in cancer cell invasion and aggressiveness in bone [136]. Inhibition of Axl in triple negative breast cancer patient-derived xenograft models led to tumor growth inhibition and significantly reduced breast cancer metastasis to bone in-vivo [137]. When Axl was inhibited in cancer cells in-vitro, there was a reduction in genes associated with epithelial-mesenchymal transition (EMT) including *SNAIL*, *VIM*, and *SLUG* [137]. Furthermore, other laboratories have shown that cancer cells can direct stromal cells, including osteoblasts, to increase autocrine Gas6/Axl signaling leading to increased cancer cell growth and survival in bone [138,139]. However, Jin, et al. demonstrated that inhibition of Gas6/Axl autocrine signaling in stromal cells abolishes paracrine signaling loops between cancer cells and stromal cells, leading to reduced cancer cell growth, and increased mouse survival [140].

**Figure 5:**
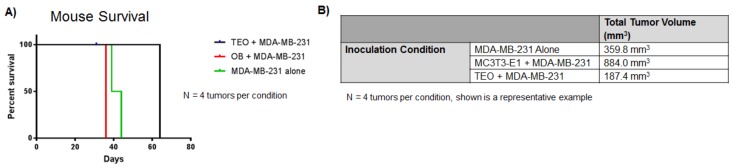
Mice inoculated with a mix of TEO cells plus MDA-MB-231 breast cancer cells lived longer and had smaller tumors than mice inoculated with a mix of untreated MC3T3-E1 cells plus MDA-MB-231 breast cancer cells, or breast cancer cells inoculated alone. Athymic nude mice were inoculated with either mixes of (1) TEO cells plus MDA-MB-231GFP breast cancer cells; (2) MC3T3-E1 osteoblasts plus MDA-MB-231GFP breast cancer cells; or (3) MDA-MB-231GFP breast cancer cells alone. (**A**) Kaplain-Meier Survival curve of percent mouse survival over time. Blue line, TEO plus MDA-MB-231GFP; red line, MC3T3-E1 plus MDA-MB-231GFP; green line, MDA-MB-231 cells alone. (**B**) Tumor size (mm^3^). At least four mice were used per condition per time point. Shown are representative examples.

Our laboratory additionally has evidence that TEO presence in tumors in-vivo leads to increased mouse time of survival and smaller tumors. When mice were injected with admixes of TEO cells plus human MDA-MB-231 breast cancer cells, mice lived, on average 20 days longer than mice inoculated with either an admix of unexposed osteoblasts plus MDA-MB-231 breast cancer cells, or MDA-MB-231 breast cancer cells alone (Figure 5A).

In addition, resultant tumors from mice inoculated with an admix of TEO cells plus breast cancer cells were nearly seven times smaller in volume when compared to tumors from admixes of unexposed osteoblasts plus breast cancer cells (Figure 5B). Furthermore, tumors composed of TEO cells were half the size of tumors formed by the injection of MDA-MB-231 cells alone (Figure 5B). These results suggest that TEO cells contribute to suppressed breast cancer cell proliferation in-vivo.

Other groups have investigated osteoblasts in the endosteal niche as contributors to prostate cancer or myeloma cell dormancy. Yumoto et al. demonstrated that co-culture of MC3T3-E1 osteoblasts plus either PC3 or DU145 prostate cancer cells resulted in a reduction of prostate cancer cell proliferation [136]. The authors found that this effect could be regulated by prostate cancer cell-derived Axl [136]. In addition, overexpression of the ligand to Axl, Gas6, increased expression of TGF-beta 2, which was found to mediate prostate cancer cell growth suppression in-vitro [136]. Furthermore, Yu-Lee and colleagues recently identified the osteoblast-derived factors GDF10 and TGF-beta 2 as a means by which prostate cancer cells are induced into a dormant state in bone [141]. When co-cultured with differentiated osteoblasts, C4-2B4, C4-2b, and PC3-mm2 prostate cancer cells were induced into a quiescent state, mediated by the proteins GDF10 and TGF-beta 2 [141]. The authors further determined that this effect occurred via activation of phospho-p38MAPK at S249/T252 sites [141]. In addition, Kobayashi et al. demonstrated that BMP7, as produced by osteoblasts and mesenchymal stromal cells, induces PC3 prostate cancer cell dormancy both in-vitro and in-vivo [142]. This effect was shown to be mediated via the p38-p21-NDRG1 signaling axis, whereby removal of BMP7, or knockdown of p21 or NDRG1 caused the cancer cells to fail to enter into dormancy [142]. Finally, when 5T33MM myeloma cells were co-cultured with either MC3T3-E1 osteoblasts or their conditioned media, myeloma cell proliferation was suppressed in-vitro [143]. These effects were in contrast to that seen when myeloma cells were treated with the conditioned media of osteoclast surrogates, which promoted myeloma cell proliferation [143]. Collectively, these results suggest that osteoblasts contribute to bone-disseminated cancer cell dormancy.

## 5. Models to Study the Effect of the Bone Niche on Cancer Cell Dormancy

Currently, there is no available model that fully recapitulates bone metastatic cancer cell dormancy as is evidenced in human disease. This is mainly due to extensive technical hurdles including, but not limited to, sustaining long-term cell growth of primary human stromal cells [144], recapitulating all steps in the tumor cell dissemination/metastatic cascade including cancer cell dormancy [145,146,147], and generation of models that permit the study of early events of initial cancer cell dissemination/seeding in bone [145,146]. Furthermore, current clinical evidence suggests that cancer cells can survive in a dormant state for decades in humans, only to be re-activated by mechanisms yet unknown [64,148,149]. There is no model available that recapitulates these events. Our laboratory and others have used several complementary approaches, including novel engineered organotypic models [144], cell lines [150,151], and extended culture bioreactors [151] to investigate cancer cell dormancy from multiple vantage points. For example, Ghajar et al. utilized 3D engineered bone marrow microvascular niches and zebrafish that contain a mutation of ectopic expression of a sprouting microvasculature to detect breast cancer cell quiescence versus growth [144]. This particular model permitted the study of early events that may occur during initial cancer cell dissemination and initiation of dormancy in a secondary microenvironment. In a clever way to overcome the technical challenges of endothelial cell growth in serum-free conditions, the authors transduced human umbilical vein endothelial cells with the gene *E4ORF1*, a human adenoviral gene that permits long-term cell survival [144]. In this way, the authors were able to successful generate a robust 3D microvasculature niche seeded with either lung fibroblasts or bone marrow mesenchymal stromal cells in order to study early interactions between weakly metastatic breast cancer cells (as surrogates for dormant cancer cells), stromal cells, and the vasculature [144]. In addition, Naumov et al. utilized D2.0R poorly metastatic mammary carcinoma cells as surrogates for dormant breast cancer cells to study breast cancer cell dissemination and dormancy in-vivo [150]. D2.0R cells were found to form metastases after extended periods of latency in the liver and other organs [150]. Thus, use of D2.0R cells permitted the study of cancer cells undergoing extended periods of latency (dormancy) in competent microenvironments in-vivo. In a later study, these same cells were used to study the growth characteristics of disseminated tumor cells upon their interaction with the extracellular matrix in-vivo [152]. Modifications to the extracellular matrix were later made, including increased deposition of type I collagen, which is frequently associated with fibrosis. These modifications then allowed the authors to study how the induction of fibrosis alters dormant cancer cell re-awakening and subsequent proliferation [153]. Finally, Sosnoski and colleagues utilized an extended, long-term bioreactor to engineer a bone-like microenvironment using mouse osteoblasts and a human metastasis-suppressed breast cancer cell line. This three-dimensional culture system permitted the authors to study long-term interactions (i.e., up to 1 year) between osteoblasts and dormant breast cancer cells [151], which is more representative of events that occur in humans. Furthermore, this model permitted the study of osteoblast-derived cytokines and their influence on dormant cancer cell growth [151]. While none of these models recapitulate the entirety of metastatic dormancy, they each shed light on important steps that occur during the process. Furthermore, use of these models permits focused study of specific elements that may contribute to cancer cell dormancy as well as early stages of the metastatic cascade.

Others have incorporated the use of specialized humanized bone microenvironments with which to study interactions between osteoblasts and breast cancer cells in cancer cell dormancy and metastasis in-vivo in an attempt to better recapitulate human disease. These methods include using irradiated human bone chip fragments that are capable of being seeded with cancer cells (including D2.0R cells as surrogates for dormant breast cancer cells [150]) plus stromal cells in Matrigel™, thus generating a partial humanized bone microenvironment for implantation in-vivo [154]. Therefore, cells used as surrogates for dormant cancer cells may be seeded directly onto intact human bones, permitting the study of dormant cancer cell interaction with a physiologically relevant human matrix in-vivo. Thus, dormant cell migration and localization within the endosteal and hematopoietic bone niches might be more accurately assessed. This method was originally developed and refined by Andreeff and colleagues [154,155]. In a similar manner, implantable 3D printed biomaterial scaffolds are currently being used to generate humanized microenvironments in-vivo [156,157]. When compared to other non-human derived 3D models, bioprinted scaffolds provide the unique advantage of mimicking both bone structure/architecture and composition. These implants can become infiltrated with a vasculature similar to human bone [158]. Bioprinted scaffolds therefore permit the study of dormant cancer cell interaction with a matrix that mimics that of bone in-vivo. Furthermore, certain groups, including the Lee group, are incorporating the use of window chambers in mice to visualize cellular processes occurring on implanted scaffolds in real-time at the resolution of single cells [158,159]. Such models may permit real-time imaging of dormant cancer cell migration, invasion, and localization within the bone microenvironment. Similar to the human bone chips, bioprinted scaffolds can also be seeded with combinations of (dormant) cancer cells plus stromal cells in Matrigel™ [159]. Both of these methods allow for the use of specific (dormant) cancer cell lines (i.e., breast vs. prostate vs. multiple myeloma) to study relevant cancer cell-stromal cell (i.e., dormant cancer cell interactions with stromal cells of the endosteal niche) interactions. Recently, Weigelt et al. compiled a review of available 3D models to study interactions between stromal cells and breast cancer cells which can be found in [145].

## 6. Dormant Cancer Cell Re-Activation in Bone

The molecular mechanisms behind exit from dormancy are not well known, but are fundamental problems in tumor biology. Exit from dormancy can be influenced by many factors, including but not limited to, secretion of tumor-promoting signals, osteoclast activation, and extracellular matrix remodeling. Recent investigations have focused on cells in the endosteal niche as contributors to cancer cell re-activation in bone. In particular, Lawson et al. found that cells in the bone microenvironment, i.e., osteoblasts and osteoclasts, are capable of either promoting (osteoblasts) or dis-engaging (osteoclasts) myeloma cells from dormancy. When treated with the conditioned media of osteoclast-like RAW264.7 cells, 5T33 MM myeloma cells exhibited increased proliferation and growth [143]. In order to determine if this effect was caused by increased osteoclastogenesis in-vivo, the authors injected soluble RANK-L, which is commonly produced by osteoblasts in-vivo [160], into myeloma disease-bearing mice [143]. Daily administration of soluble RANK-L resulted in increased osteoclastogenesis and subsequent increased bone resorption. Interestingly, the authors observed that there was a reduction in the number of dormant myeloma cells in the bone upon increased bone resorption [143]. These results suggest that myeloma cancer cell re-activation is initiated by increased osteoclast activity as mediated by osteoblast-derived factors in bone. In a separate investigation examining mechanisms of dormancy in prostate cancer cells, reduction in osteoblast expression of TGF-beta and Gas6 led to a release from dormancy of PC3 and DU145 prostate cancer cells [136]. Furthermore, in a study by Kobayashi and colleagues, reduction of osteoblast- and mesenchymal stromal cell-derived BMP7 led to increased PC3 prostate cancer cell growth both in-vitro and in-vivo [142]. Therefore, these data suggest that osteoblasts are a key source of proteins, including RANK-L, TGF-beta, Gas6, and BMP7, that regulate dormant cancer cell re-activation in bone.

Additional studies have shown that direct interaction with osteoblasts and cells of the osteogenic niche reduce periods of breast cancer cell latency and promote cancer cell growth. In an investigation by Wang et al., when MCF-7 or MDA-MB-361 breast cancer cells were injected via intrailiac injection into mice, initial cancer cell colonization was slow for the first 2–4 weeks (MCF-7) or 4 months (MDA-MB-361) post-injection as evidenced by a lack of Ki67 staining and retention of an H2B-GFP label [161]. These results indicate a period of latency for the cancer cells, as defined as a “pre-osteolytic stage.” However, upon direct interaction both in-vitro and in-vivo with either mouse MC3T3-E1 osteoblasts, human FOB1.19 fetal osteoblasts, or human mesenchymal stromal cells (in-vitro); or alkaline phosphatase, collagen-I, RUNX2, or osterix positive osteogenic niche cells (in-vivo), there was a statistically significant increase in breast cancer cell proliferation [161]. Proliferation effects were less pronounced when breast cancer cells were separated from the osteogenic cells using a Boyden Chamber assay. Additional experiments revealed that the osteogenic cells were interacting with the breast cancer cells via N-cadherin and E-cadherin heterotypic adherens junctions, and that these interactions promoted breast cancer cell proliferation and escape from a latent, pre-osteolytic phase [161]. In fact, mice inoculated via intrailiac injection with MCF-7 cells, then treated with anti-E cadherin exhibited reduced cancer cell proliferation and less bone lesions [161]. Therefore, these results suggest that direct interaction with osteoblasts and osteogenic cells, such as mesenchymal stromal cells, may be one way in which latent breast cancer cells are released from dormancy in bone.

We would be remiss to not discuss an interesting study carried out by Gao and colleagues. Upon injection of 4T07 breast cancer cells into the tail veins of mice, 4T07 cells infiltrated the lung, however remained quiescent and appeared solitary in number as evidenced in lung sections [162]. However, when 4T07 cells were engineered to express Coco, a secreted inhibitor of TGF-beta, and injected into mice, single cells began to proliferate in the lung approximately 14 days post injection, such that by day 35, macrometastic lesions had formed in the lungs. These results suggest that Coco induces dormant cancer cell reactivation in the lung. Additional analyses demonstrated that reactivation of dormant breast cancer cells in the lung is caused by inhibition of BMP ligands via Coco [162]. Since bone osteoblasts highly express BMP ligands, it was next investigated whether this same phenomenon is found in bone. Interestingly, when Coco was silenced in 4T1 breast cancer cells that were inoculated via intracardiac injection into mice, no change was seen in cancer cell growth or lesion formation in bone [162]. These results are intriguing, given that BMP levels are typically high in bone, and BMPs (especially BMP-2) are required for osteoblast differentiation [163,164]. To further explore this result, the authors examined bone production of phospho-Sma-And Mad-Related Protein (also known as phospho-Mothers Against Decapentaplegic Homolog, or phospho-SMAD), a factor in the BMP-2/Smad signaling pathway [165]. While cells in the growth plate exhibited strong nuclear staining of phospho-SMAD, metastatic lesions detected in the bone were phospho-SMAD negative, which indicates that the lesions originated from phospho-SMAD negative cancer cells. By contrast, more than 94% of Coco responsive lesions in the lung were phospho-SMAD positive [162]. Therefore, these results suggest that while BMP signaling as mediated by osteoblasts in the bone may elicit dormant prostate cancer cell re-activation [142], mechanisms governing the process of cancer cell re-activation (A) may be dependent on the organ from which disseminated tumor cells originate (i.e., breast versus prostate), or (B) there may be subsets of dormant cancer cells not responsive to engagement of BMP proteins, and thus incapable of BMP-mediated re-activation. In the case of (B), this may suggest that dormant cancer cell re-activation is governed by multiple mechanisms, thus favoring a multi-modal clinical treatment approach to preventing indolent cell re-activation in cancer patients.

## 7. Osteoblasts in the Early Stages of Cancer Metastasis to Bone

There is no model currently available that fully recapitulates the entirety of human metastasis to bone [155]. Efforts to study the early stages of disease progression, especially events that occur during initial cancer cell seeding in bone, have been hampered by lack of available mouse model systems, especially immuno-competent models, technical limitations, and lack of cell lines available that recapitulate all steps of disease progression [166]. As a result, our knowledge of early events of cancer metastases to bone is limited at best. A survey of the available literature revealed only two publications specifically dedicated to exploring the role of osteoblasts in early stage bone metastatic breast cancer [167,168]. Of these two, Bodenstine et al. sought to elucidate mechanisms that modulate osteoblast populations during early stage breast cancer bone metastasis [167]. The authors co-injected pre-osteoblast cells plus bone metastatic breast cancer cells via intratibial injection into mice and assessed tumor growth at 5 and 6 weeks post-inoculation. By 5 weeks post-inoculation, mice co-injected with osteoblasts plus breast cancer cells exhibited large, palpable tumors, that extended into the extra-osseous space, and which grew slightly by week 6 [167]. By comparison, tumors that formed in mice injected with cancer cells alone were half the size of those co-injected with osteoblasts, and remained contained in the bone. Osteolysis was found to occur in all treatment groups as exhibited by TRAP staining. The authors concluded that there is an important interaction between bone osteoblasts and metastatic breast cancer cells as evidenced by a dramatic increase seen in tumor size and growth into the extra-osseous space as compared to cancer cells injected alone [167]. While the time points studied and mouse models used may not be fully reflective of early events, they help shed light on communication that occurs between breast cancer cells and osteoblasts in bone. In the second publication, Scimeca et al. used patient samples consisting of breast infiltrating carcinomas, benign breast lesions, and bone metastatic lesions to identify the appearance of breast osteoblast-like cells in primary mammary lesions as an early predictor of breast cancer metastases to bone [168]. The authors found that there were a large number of breast cancer cells that underwent EMT in primary infiltrating carcinomas. This corresponded to a large number of breast-osteoblast-like cells found in the primary carcinomas that were positive for expression of RANK-L and vitamin D receptor. Intriguingly, breast osteoblast-like cells were also located in matched bone metastases. These results further suggest that crosstalk occurs between bone osteoblasts and metastatic breast cancer cells that may occur as early as when breast cancer cells are located in the primary site [168].

Wang et al. additionally carried out a study in an attempt to determine the involvement of osteoblasts in the early stages of bone metastatic prostate cancer [65]. The authors surmised that the distribution of cells of the osteoblast lineage may not be uniform within the bone, and that this may have important implications for cancer cell homing and adhesion during prostate cancer cell bone metastasis. This was in response to a study carried out by the Taichman group, suggesting that metastatic prostate cancer cells target hematopoietic stem cell niches in bone and compete with hematopoietic stem cells for development of prostate cancer bone metastases [169,170]. As per the Taichman group, the role of the osteoblast, in this case, was to maintain the hematopoietic stem cell niche as a reserve population in early stages of prostate cancer metastases to bone [169,170]. By way of the Wang study, mice were injected via intracardiac inoculation with bone-tropic prostate cancer cells. It was found that the prostate cancer cells preferentially homed to areas of the lateral endocortical bone regions, which were associated with 5-fold higher the number of bone osteoblasts compared to medial endocortical regions [65]. The authors also determined that the SDF-1/CXCR4 signaling axis was key to prostate cancer cell homing to these regions, as osteoblasts express the SDF-1 ligand and prostate cancer cells express the CXCR4 receptor. Use of the CXCR4 inhibitor AMD3100 disrupted the preferential homing of prostate cancer cells to the lateral endocortical region of bone [65]. Thus, these results suggest that prostate cancer cells preferentially home to osteoblast-rich areas in early stages of the disease.

## 8. The ‘Vicious Cycle’ of Cancer Metastasis to Bone

**Figure 6:**
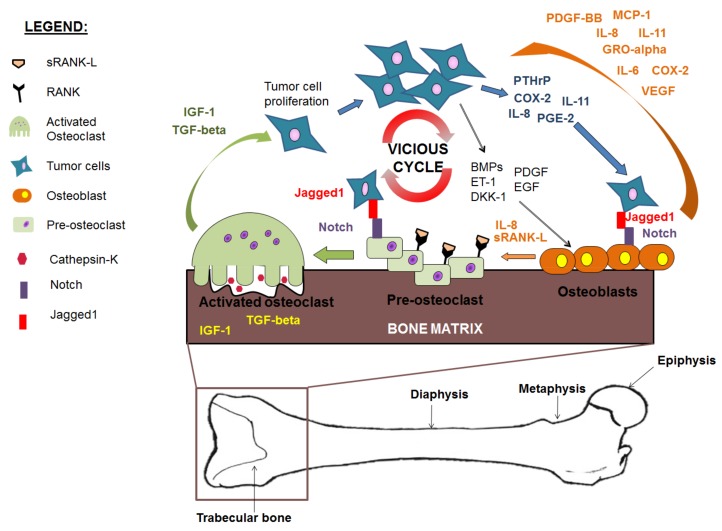
The ‘Vicious Cycle’ of bone degradation. In the ‘vicious cycle’ of cancer metastasis to bone, metastatic cancer cells produce parathyroid hormone related protein (PTHrP), interleukin-8 (IL-8), interleukin-11 (IL-11), prostaglandin E2 (PGE2), and cyclooxygenase-2 (COX-2) (lytic and blastic cancers) as well as bone morphogenic proteins (BMPs), endothelin-1 (ET-1), dickkopf-1 (DKK-1), platelet-derived growth factor (PDGF), and epidermal growth factor (EGF) (blastic cancers). These factors promote increased osteoblast production of interleukin-6 (IL-6), IL-8, monocyte chemoattractant protein-1 (MCP-1), vascular endothelial growth factor (VEGF), growth related oncogene-alpha (GRO-alpha), IL-11, and COX-2 that act as chemoattractants, maintenance, and survival factors for lytic metastatic cancer cells. Growth factors, including VEGF, insulin growth factor-1 (IGF-1), platelet-derived growth factor-BB (PDGF-BB), and tumor-growth factor-beta (TGF-beta) as produced by osteoblasts serve to promote proliferation of blastic cancers. Osteoblasts also produce increased amounts of soluble receptor activator of nuclear factor kappa beta-ligand (RANK-L), which binds to RANK receptors on pre-osteoclasts to initiate osteoclastogenesis. Furthermore, breast cancer cells that express Jagged1 ligand bind to the Notch receptor on osteoblasts leading to increased osteoblast-derived RANK-L expression and increased osteoclast activation. Jagged1 expressing breast cancer cells can also bind to the Notch receptor on pre-osteoclasts and initiate osteoclastogenesis independent of RANK-L. Activated osteoclasts resorb bone by secretion of proteases including cathepsin K into resorption pits, and release TGF-beta and IGF-1 from the bone matrix. TGF-beta and IGF-1 act on metastatic tumor cells to promote proliferation and continued expression of PTHrP, IL-8, IL-11, PGE2, COX-2, BMPs, ET-1, DKK-1, PDGF, and EGF from cancer cells.

Cancer metastases to bone disrupt the tightly regulated balance between osteoblasts and osteoclasts. Osteoblasts, in particular, unknowingly play important roles in fueling both (1) tumor cell growth and (2) sustained osteoclastogenesis and bone resorption. In a classic model of osteolytic metastasis proposed by Guise and Mundy, osteoblasts are stimulated to overproduce RANK-L by PTHrP as produced by breast cancer cells [84,171,172]. Osteoblast-secreted RANK-L binds the RANK receptor on osteoclast precursors, inducing osteoclast differentiation. Activated osteoclasts, in turn, secrete cathepsin K and other cysteine proteinases into the bone matrix, which help to degrade type I collagen, and ultimately result in bone matrix breakdown [173,174]. In addition, TGF-beta and IGF-1, released from the bone matrix, stimulate the cancer cells to produce additional PTHrP [28,175]. Furthermore, in addition to RANK-L dependent pathways, there is additional evidence to support RANK-L independent osteoclastogenesis via cancer-derived IL-8 binding to IL-8 receptors on pre-osteoclasts [176]. In fact, Bendre et al. suggested that breast cancer-derived IL-8 may act earlier in the vicious cycle than tumor-derived PTHrP [177]. As a result of constitutive osteoclast activation and an inability of osteoblasts to lay down bone matrix, sustained bone degradation occurs [178,179]. This feedback establishes a ‘vicious cycle’, resulting in continued production of osteoclast initiating factors by osteoblasts, and constitutive activation of osteoclasts and osteolytic cancer cells (Figure 6).

Furthermore, similar to the ‘vicious cycle’ of osteolytic bone destruction in bone metastatic breast cancer, a similar mechanism has been described for occurrences in multiple myeloma. Multiple myeloma cells have been shown to produce factors that inhibit osteoblast differentiation and growth, including IL-3, TNF-alpha, and TGF-beta [180,181]. At the same time, multiple myeloma cells also produce factors that stimulate osteoclastogenesis including RANK-L, IL-6, MIP-1 alpha, and TNF-alpha [180,182,183,184,185]. Moreover, suppressed osteoblast activity also results in a reduction of osteoblast-derived OPG, a decoy receptor for RANK-L. The reduction in osteoblast bone deposition and increase in osteoclast bone resorption lead to sustained bone destruction which promotes disease progression [186].

In bone metastatic prostate cancer, similar mechanisms occur to that of lytic disease, however lesions that form are blastic due to sustained bone deposition. Metastatic prostate cancer cells produce increased amounts of endothelin-1 which causes sustained osteoblast differentiation [121]. In addition, DKK-1, a Wnt antagonist, is suppressed leading to increased osteoblast differentiation and proliferation [121]. Activated osteoblasts then produce increased amounts of growth factors including VEGF, IGF-1, and TGF-beta, which support cancer cell growth and proliferation [93,116]. Sustained bone deposition occurs at the same time as bone resorption [118] (Figure 6). Osteoblast bone matrix is laid down in a disorganized fashion, resulting in poorly developed, weak bone [114].

In years since the first description of the ‘vicious cycle’, several groups have added additional components to the classical model. Among them, Sethi et al. demonstrated that Jagged1, as expressed on tumor cells, can interact with both osteoblasts and pre-osteoclasts in the bone niche to further drive sustained bone resorption [163]. Specifically, Jagged1 was shown to bind to the receptor Notch on osteoblasts, leading to signaling through Rbpj and Hey1, and subsequent increased expression of osteoblast-derived IL-6 [60]. IL-6 in the tumor microenvironment has been demonstrated to be a potent driver of tumor cell growth [187]. In addition to binding Notch on osteoblasts, Jagged1 can also bind the Notch receptor on pre-osteoclasts, leading to increased osteoclastogenesis and the formation of mature osteoclasts capable of resorbing bone. Increased bone resorption by osteoclasts was found to release increased amounts of TGF-beta from the bone matrix, thus promoting tumor growth and survival [188].

Our group additionally found that IL-6, IL-8 (murine MIP-2), GRO-alpha (murine KC), MCP-1, and VEGF as produced by osteoblasts and found in their conditioned media enhanced osteoclast formation [50]. Importantly, IL-6, as administered to pre-osteoclasts in concentrations representative of that found in osteoblast conditioned media, led to the formation of TRAP+ osteoclasts [50]. Furthermore, IL6, IL-8, MCP-1, GRO-alpha, and VEGF were found to be maintenance and survival factors for bone metastatic breast cancer cells [50]. Therefore, osteoblast secretion of IL-6, IL-8, MCP-1, GRO-alpha, and VEGF contributes to osteoclast bone resorption independently of RANK-L in the classic ‘vicious cycle’ model, as well as promotes breast cancer survival in bone.

Furthermore, cyclooxygenase-2 (COX2) as expressed by both osteoblasts [204] and breast cancer cells [199] has been demonstrated to facilitate the formation of a microenvironment favorable for cancer cell growth and survival. In a study by Singh, et al. overexpression of breast cancer cell-derived COX2 led to an increase in cancer cell invasion and metastasis in a mouse model of breast cancer metastasis to bone [199]. Increased COX2 lead to increased expression of both PGE2 and IL-8 in breast cancer cells, and especially breast cancer cells with bone tropism [196]. Moreover, COX2 was found to mediate the production of interleukin-11 (IL-11), up to a 6-fold increase, in triple negative, ER+, and bone-seeking metastatic breast cancer cells [206]. Since both IL-11 and IL-8 as produced by both osteoblasts [50,193] and breast cancer cells [177,197] have been shown to stimulate osteoclastogenesis both by RANK-L dependent and independent mechanisms [176,177,197,221], a system favoring increased COX2 expression would lead to increased osteolytic metastases and thus further drive the ‘vicious cycle’ of breast cancer bone metastases. These findings as a whole have been summarized in Figure 6 and Table 1.

## 9. Osteoblasts in Advanced Stage Metastatic Disease 

There has been extensive evidence in recent years demonstrating the role of osteoblasts in late stage bone metastatic cancers. By way of crosstalk with bone metastatic cancer cells, osteoblasts are diverted from synthesizing bone matrix to producing proteins that play vital roles in cancer cell attraction [128,129], maintenance [50], and survival [6,130,131] during cancer progression in bone. Specifically, we showed that osteoblasts are a key source of cytokines necessary for metastatic breast cancer cell maintenance and survival in bone. When either co-cultured with MDA-MB-231 metastatic breast cancer cells or treated with their conditioned media, we found that osteoblasts increased their production of the inflammatory cytokines IL-6, IL-8, and MCP-1 [51]. It is well documented that these cytokines are capable of initiating osteoclastogenesis, as well as are capable of increasing the activity of osteoclasts [176,177,196,222,223,224]. In a follow-up study, we additionally found that osteoblasts were key sources of VEGF and GRO-alpha (mouse KC) when in the presence of metastatic breast cancer cells [50]. Similar results were seen when we examined both early (10 days) and late (20 day) differentiated osteoblasts, although effects were more pronounced (higher statistical significance) upon metastatic breast cancer cell co-culture or conditioned media treatment with late differentiated osteoblasts (20 days differentiated) [50]. Most importantly, we found that, in comparison to differentiated osteoblasts, breast cancer cells expressed negligible amounts (~3 pg/mL) of MCP-1, a chemokine involved in inflammation [225]. Osteoblasts, however, expressed comparably larger amounts of MCP-1 (~2 ng/mL), which was increased up to 7 fold upon treatment with breast cancer conditioned medium [50]. MCP-1 has been demonstrated as being important for cancer cell proliferation, migration, and invasion [226,227,228,229]. Thus, these results imply that bone metastatic breast cancer cells rely on osteoblast production of MCP-1 as a key factor for their oncogenic properties in bone. In addition, when MDA-MB-231 metastatic breast cancer cells were inoculated via intracardiac injection into athymic nude mice, we observed an increase in bone-derived cytokine production in disease-bearing mice [50]. Interestingly, we found that the increase in bone cell-derived cytokine production was the most pronounced in the metaphyseal ends, as opposed to the diaphysis, of murine femurs [50]. Immunochemistry revealed that the osteoblast-derived cytokines VEGF and MCP-1 were specifically localized to bone ends composed of trabecular bone and not the bone shaft in non-cancer bearing mice [130]. IL-6 was found to be localized to the bone marrow [130]. Importantly, osteoblast conditioned media was a potent chemoattractant for metastatic breast cancer cells and also elicited the formation of TRAP+ osteoclasts [50]. Thus, these results demonstrate that breast cancer cells utilize osteoblast-derived cytokines to facilitate breast cancer cell colonization and survival in the bone microenvironment. These results additionally show that the osteoblast-derived cytokines IL-6, MCP-1, IL-8 (mouse MIP-2), GRO-alpha (mouse KC), and VEGF are also capable of stimulating osteoclastogenesis either in addition to or in the absence of the RANKL-RANK pathway [50]. Our findings additionally extend the ‘vicious cycle’ of bone degradation to include the cytokines IL-6, MCP-1, IL-8 (mouse MIP-2), GRO-alpha (mouse KC), and VEGF as produced by osteoblasts [50] (Figure 6). In addition to these effects, we found that osteoblasts had altered morphology, exhibited decreased expression of alkaline phosphatase, osteocalcin, and bone sialoprotein in-vitro, as well as underwent apoptosis in-vivo when adjacent to bone metastatic breast cancer cells in situations of advanced tumor burden [28,179,230].

In addition to effects in bone metastatic breast cancer, it has been demonstrated that osteoblasts and osteoblast-derived factors also have metastasis-promoting effects in bone metastatic prostate cancer. In a study by Karlsson and colleagues, osteoblast-derived TGF-beta, among other factors, was shown to increase PC-3U prostate cancer cell migration as well as formation of pro-migratory protrusions [129]. In a series of experiments by the Taichman group, osteoblast-derived CXCL12 (SDF-1) was found to mediate bone metastatic prostate cancer progression via binding of its receptor CXCR4 on the prostate cancer cells. CXCL12 was additionally found to be highly expressed in the skeleton, and was localized to the ends of long bones; areas to which bone metastatic cancer cells preferentially traffic [62,128,231,232]. In addition, Lee et al. determined that osteoblast expression of c-met and VEGFR2 promoted PC-3 and C4-2B prostate cancer cell growth in bone, as well as increased osteoclastogenesis and osteolytic lesion formation [131]. Inhibition of osteoblast-derived c-Met and VEGFR2 or mouse treatment with cabozantinib, a tyrosine kinase inhibitor with a high affinity for c-Met and VEGFR2 [190], led to a reduction in bone metastatic prostate tumor growth, and also reduced osteoblast production of RANKL and M-CSF, two factors necessary for osteoclastogenesis [131]. On the other hand, another group determined that treatment with cabozantinib may in fact lead to osteoblast upregulation of factors that promote bone metastatic prostate cancer cell migration and survival. Primary mouse osteoblasts were treated with 100 nM of cabozantinib and their proliferation and differentiation analyzed. It was found that while treatment with cabozantinib led to a decrease in osteoblast proliferation, osteoblast differentiation, as measured by alkaline phosphatase and osteocalcin expression, as well as osteoblast mineralization were significantly increased [233]. Further analysis revealed that increased osteoblast differentiation led to an increase in the osteoblast-derived factors IGFBP2 and WNT16, among others [233]. These factors have been shown in the literature to stimulate anchorage independent cancer cell growth [234], and have been linked to therapeutic resistance in cancer [235]. When PC3-mm2 or C42B4 prostate cancer cells were treated with the conditioned media of cabozantinib treated osteoblasts, there was an increase in anchorage independent prostate cancer cell growth as well as an increase in prostate cancer cell migration [233]. Overall, these data suggest that factors produced by osteoblasts promote metastatic prostate cancer progression in bone.

A similar role has recently emerged for osteoblasts in the progression of multiple myeloma in bone. In a study by van Andel et al., it was discovered that osteoblasts express R-spondins which promote Wnt signaling in an leucine rich repeat containing G protein-coupled receptor-4 (LGR4)-dependent manner in multiple myeloma cells [236]. Multiple myeloma cells were found to have aberrant LGR4 signaling, and thus were capable of increased sensitivity to Wnt signaling [236]. Incidentally, activation of the canonical Wnt signaling pathway in multiple myeloma cells helped to drive cancer cell proliferation, and was associated with increased cancer cell dissemination to bone, increased disease progression, and increased therapeutic resistance [237,238]. In addition, Nemani et al. determined that multiple myeloma cells initiate osteoblasts to reduce their expression of decorin, a extracellular matrix proteoglycan with tumor suppressive properties [239]. Multiple myeloma induced suppression of decorin in osteoblasts was found to be mediated by chemokine (C-C motif) ligand-3 (CCL3). When decorin was reduced in osteoblasts, this promoted multiple myeloma cell proliferation and survival [239].

Clinically, patients with osteolytic metastases are treated with drugs that block osteoclast activity. Therapies utilizing drugs called bisphosphonates, such as pamidronate, prevent osteoclast bone resorption by inducing osteoclast apoptosis. Pamidronate binds to and adsorbs to hydroxyapatite crystals in the bone matrix. Osteoclasts ingest the drug as they degrade bone. Farnesylation and geranylgeranylation of proteins necessary for osteoclast function are inhibited, and osteoclasts undergo apoptosis [240]. Pamidronate does not inhibit bone mineralization or formation [240]. By reducing osteoclast-mediated bone resorption, pamidronic acid decreases the rate of bone turnover, stabilizes the bone matrix, and reduces hypercalcemia. While pamidronate is a treatment for bone metastatic breast cancer by blocking osteoclast activity, it is not curative [91,241]. Lesion progression is slowed, but pre-existing lesions do not heal [92,178,242,243]. There are currently no treatments that specifically target osteoblasts to reduce osteoblast apoptosis or promote bone deposition. Severe bone pain, fractures, hypercalcemia, and spinal cord compression may also occur due to sustained bone resorption [83,84,244,245]. The inability of bone to regenerate following bisphosphonate treatment suggests that bone metastatic breast cancer cells alter osteoblast function in addition to constitutive activation of osteoclasts.

There has, however, been encouraging data recently examining use of the tyrosine kinase inhibitor, cabozantinib, as an indirect anti-tumor agent and direct modulator of osteoblasts. Several groups have recently demonstrated that cabozantinib is capable of specifically targeting bone osteoblasts to decrease their production of RANK-L and increase their production of the RANK-L decoy receptor OPG. As a direct result of altered ratio of RANK-L/OPG, osteoclastogenesis decreased and osteoblast numbers increased compared to control [246,247]. In mice, administration of cabozantinib reduced prostate cancer cell proliferation in bone and induced prostate cancer cell apoptosis [248]. A different study also revealed that tumor growth was suppressed, along with a reduction in the formation of bone-resorbing osteoclasts via a decrease in osteoblast-derived RANKL and M-CSF in a mouse model of bone metastatic prostate cancer [131]. Intriguingly, however, use of cabozantinib in patients with advanced metastatic castration-resistant prostate cancer as part of the COMET-1 and COMET-2 phase III clinical trials did not extend overall patient survival as compared to the treatments prednisone or prednisone plus mitoxantrone [249]. Over 1000 patients were followed as part of this analysis [249]. A small benefit from cabozantinib was seen, however, towards improvement in bone scan response, radiographic progression-free survival, and reduction in symptomatic skeletal events in patients with advanced metastatic castration-resistant prostate cancer as part of the COMET-1 trial [250]. Furthermore, patient treatment with cabozantinib led to increased expression of alkaline phosphatase and cross-linked type I collagen, suggesting increased osteoblast differentiation and bone matrix organization [250]. These results were similar to in-vitro studies which showed that primary mouse osteoblast treatment with cabozantinib resulted in increased osteoblast expression of alkaline phosphatase [233].

As previously mentioned, bisphosphonate drug therapy does not cure osteolytic lesions. One possibility for this occurrence is that breast cancer cells affect osteoblast differentiation. Osteoblasts develop from osteoprogenitor cells, which undergo a program of differentiation to become mature, functional osteoblasts capable of synthesizing bone matrix [21]. Work by Mercer et al. demonstrated that culturing mouse osteoblasts with conditioned medium from a human metastatic breast cancer cell line inhibited expression of osteoblast differentiation markers and completely blocked the ability of osteoblasts to mineralize bone matrix [179]. Since osteoblasts do not differentiate properly in the presence of breast cancer cells, it is possible that the cancer cells may alter the overall protein secretion profile of osteoblasts. This alteration may prevent osteoblasts from producing the differentiation proteins necessary for developing into mature, bone-depositing cells, as well as by inducing production of cytokines that contribute to the progression of bone metastasis, increase activation of osteoclasts, and contribute to the formation of osteolytic lesions.

## 10. Osteoblast Alignment and Bone Matrix Organization Are Altered by Direct Interaction with Bone Metastatic Cancer Cells

In recent years, there have been a number of reports demonstrating correlations between bone matrix organization, strength, and the involvement of bone osteoblasts. A recent report by Matsugaki et al. demonstrated the importance of the alignment of osteoblasts in the bone matrix toward preserving tissue microstructure [115]. When compared to a control surface, the authors determined that, on an abnormally arranged surface, osteoblasts aligned themselves orthogonal to the direction of artificial matrix. This orientation caused the osteoblasts to form elongated f-actin stress fibers perpendicular to matrix collagen stress fibers and crystals of biological apatite [115]. Incidentally, this abnormal orientation of osteoblasts, and associated disorganized matrix, directly reflects that seen in tumor-bearing bones. In fact, direct interaction with bone metastatic prostate, breast, or multiple myeloma cells led to disorganized arrangement of osteoblasts along with subsequent disruption of the alignment of collagen fibers and biological apatite in the long bones of tumor-bearing mice [114,251]. This effect was not seen when osteoblasts were treated with cancer cell conditioned medium, suggesting that soluble cancer-derived factors play no role in modulating osteoblast orientation within the tumor microenvironment [251]. Furthermore, during co-culture with cancer cells, osteoblasts increased their production of cadherin-11, a protein that promotes cell-cell and cell-substrate adhesion as well as regulates collagen synthesis [252,253], and connexin-43 [251], a ubiquitous connexin that functions in the formation of gap junctions [254]. These results suggest that alteration of osteoblast alignment in the bone matrix upon direct interaction with bone metastatic cancer cells may be mediated by gap junction signaling or cell-cell adhesion molecules.

Osteoblast arrangement within bone determines the bone matrix microstructure, including alignment of both biological apatite and collagen fibers [114,115,251]. It has been determined that alignment of biological apatite is strongly correlated to Young’s modulus, or the ability of a material to withstand changes in longitudinal compression or tension [255]. In addition, collagen fiber orientation in bone was correlated with bone toughness [256]. Importantly, loss of mechanical function within bone is associated with malalignment of the bone matrix, contributing to diseases such as osteoporosis and cancer-induced bone disease [257]. Thus, as a whole, these results suggest that increased malalignment of osteoblasts, as a result of direct interaction with bone metastatic cancer cells, causes disrupted formation of the bone matrix, including disorganized arrangement of biological apatite and collagen fibers, and reduced mechanical function. Moreover, reduced bone mechanical function correlates with both reduced bone toughness and reduced capacity to withstand longitudinal forces of tension or compression [114]. These structural changes further contribute to overall bone degradation in bone metastatic cancers.

## 11. Cancer Cells Are Capable of Mimicking Osteoblasts in the Tumor Microenvironment

In addition to cancer cells modifying osteoblast functions, one of the most devious actions cancer cells can take is that of osteomimicry. In order to better survive in the bone microenvironment, bone metastatic cancer cells pay osteoblasts the ultimate form of flattery in an attempt to resemble a resident osteoblast bone cell. There are documented reports of bone metastatic cancer cells producing normal bone turnover markers such as alkaline phosphatase, and markers of bone remodeling such as MMPs and collagen [258,259,260]. To make matters worse, the cancer cells produce increased amounts of factors that fuel bone resorption, including MMPs, which further contribute to the ‘vicious cycle of bone degradation’ [261]. Tan and colleagues conducted an interesting study to determine ways breast cancer cells may be transformed into osteoblast-like cells in the bone. After undergoing an epithelial-mesenchymal transition, as stimulated by the conditioned media from cancer-associated fibroblasts, MCF-7 and T47D breast cancer cells were then stimulated with BMP2 to induce an osteoblast-like phenotype [259]. Compared to untreated breast cancer cells, treated breast cancer cells increased their expression of osteoblast cadherin CDH11, RUNX2, osteonectin (SPARC), the bone matrix-remodeling protein periostin, as well as a defined set of “bone-related genes”. These effects were not observed in untreated breast cancer cells [259]. These results suggest that breast cancer cells are capable of undergoing osteomimicry after EMT, and express factors that are master mediators of bone remodeling signaling pathways typically found in osteoblasts. Furthermore, a study by Hagberg Thulin et al. revealed that treatment with osteoblast conditioned media increased LNCaP-19 and LNCaP prostate cancer cell expression of osteoblast-like genes, including *RUNX2*, *MMP2*, *CDH11*, *osteonectin*, and *osteopontin* [260]. Furthermore, in a study by Hassan et al., it was demonstrated that upon differentiation, osteoblasts normally express microRNA-218 in the bone microenvironment [258]. MiR-218 was found to have potent osteogenic properties, including enhancement of osteoblast differentiation and mineralization, as well as increased osteoblast lineage commitment of bone marrow progenitor cells. Interestingly, miR-218 expression in metastatic breast cancer cells leads to abnormal expression of genes associated with osteomimicry which promote cancer cell homing to bone, including the chemokine receptor *CXCR4* [258]. Moreover, treatment with miR-218 also increased bone metastatic breast cancer cell expression of genes associated with normal osteoblast bone turnover including *bone sialoprotein* and *osteopontin* [258]. Finally, Graham et al. demonstrated that PC-3 and C4-2B bone metastatic prostate cancer cell lines have enhanced bone invasive properties as compared to LNCaP cells [262]. PC-3 and C4-2B enhanced bone metastatic potential was found to be due to increased cancer cell expression of the osteoblast-like factors type IA collagen, osteocalcin, and osteopontin. These effects could be enhanced in the PC-3 and C4-2B prostate cancer cells by BMP-2 [262]. Thus, these results as a whole suggest that osteomimicry, or acquisition of an osteoblast-like phenotype, may be a way that cancer cells specifically traffic to the bone microenvironment and further create a suitable secondary niche supportive of cancer cell growth and survival.

## 12. Future Questions to Be Considered

Even though substantial progress has been made in the past decade towards understanding metastatic cancer cell progression in bone, many questions remain. It is clear that bone metastatic cancer cells hijack endogenous cells of the bone and exploit normal processes in order to favor cancer cell maintenance and survival. What is the mechanistic ‘tipping point’ that permits switching from ‘normal’ bone remodeling processes to promoting cancer progression? As previously discussed, Lawson et al. found that disseminated cancer cell entry into dormancy may be a reversible state, and that promotion of dormancy may occur via interactions with osteoblasts and cells of the osteogenic lineage [143]. Exit from dormancy was postulated to occur by function of increased osteoclast bone resorption of the niche, suggesting that better understanding of interplay between dormant cancer cells and the molecular mechanisms involved in active bone remodeling may be keys to controlling entry and exit from dormancy. More importantly, if osteoblasts are capable of inducing cancer cell dormancy in early stages of the disease, is it possible for that capability to be exploited in order to induce a perpetual state of cancer cell proliferative quiescence in bone? To that point, Wang et al. demonstrated that prostate cancer cells preferentially seed in osteoblast-rich areas of bone, and in some cases were found in direct contact with osteoblasts, especially in lateral endocortical regions of bone as opposed to medial regions [65]. This was primarily evident in the early stages of cancer cell homing to bone; however the authors noted that preferential seeding of osteoblast-rich areas also occurred at later times of up to 3 weeks in a mouse model of intracardiac injection [65]. We also demonstrated a similar trend of metastatic breast cancer cell seeding in bone, whereby metastatic breast cancer cells injected into mice via the intracardiac route preferentially homed to and seeded in (1) the metaphyseal regions of long bones, areas of high bone turnover, and (2) the endosteal marrow of long bones are opposed to central marrow [62]. These data not only emphasize the importance of bone remodeling in cancer cell homing, seeding, and dormancy in bone, but also that osteoblasts, in particular, might be one of the keys to control of these processes.

What event(s) cause osteoblasts to switch from “dormancy-promoting” to “metastasis-promoting”? While Lawson et al. described that increased osteoclast activity and bone resorption might be one way in which cancer cells exit dormancy [143], it is unclear how cancer cells are capable of remaining in a dormant state for decades even though there is routine bone turnover. Is it necessary for an adverse event on a larger scale, such as a fracture, or perhaps osteopenia, to occur before exit from dormancy via bone remodeling can occur? Similarly, might it be that exit from dormancy can only occur in situations where there may be a temporary increase in bone resorption in excess of bone deposition? Do these changes need to local (as in the case of a fracture) versus systemic (as in the case of osteopenia) in bone to elicit escape from dormancy via bone remodeling? These questions have yet to be elucidated. Furthermore, are there specific osteoblast- or bone-derived biomarkers that may be capable of indicating which patients harbor dormant cancer cells in their bones? In light of data provided by Wang et al. [65], these markers may include alterations in osteoblast markers such as alkaline phosphatase, BMPs, or even master regulators of osteoblast function such as RUNX2. And, are there biomarkers to indicate which patients will progress to metastatic disease prior to the formation of occult macrometastatic lesions? Again, based on evidence by Lawson, the answer to this question may be, in part, directly related to the balance between bone deposition and bone resorption, whereby sustained bone resorption marked by elevated levels of calcium and growth factors stored in bone such as TGF-beta and IGF-1 might be early indicators of disease advancement [143]. If biomarkers are discovered to indicate dormant bone disease, might there be a method to attack and eliminate sleeping cancer cells in bone prior to the development of occult lesion formation? And, might there be a way to sensitize dormant cancer cells to chemotherapeutic treatments? Further investigations are needed to study the intricacies of bone remodeling, including direct interplay between osteoblasts, osteoclasts, and cancer cells, in order to elucidate mechanisms of crosstalk in cancer cell dormancy and progression in bone.

## 13. Conclusions

The bone microenvironment is a fertile ‘soil’ for metastatic cancer cells (the ‘seeds’) and it is evident that the ‘soil’ is extremely influential in facilitating cancer cell ‘seed’ growth. Crosstalk between cells of the bone, especially the bone osteoblasts, and cancer cells drives cancer cell progression and metastasis in the bone microenvironment. It is becoming increasingly evident that osteoblasts in the bone are major regulators of cancer cell bone progression and metastasis. Osteoblasts clearly play key roles in functioning as ‘tumor-suppressors’ versus ‘tumor-promoters’ during different stages of bone metastatic cancer. Indeed, it is intriguing to consider that osteoblasts may possess this dual role during cancer cell progression in bone. Furthermore, as of yet, there have been no data to describe what mechanisms govern the balance between osteoblast ‘dormancy-promoting’ versus osteoblast ‘metastasis-promoting’ functions. The available data currently point towards bone-disseminated cancer cells as master manipulators to this process. However, might it be possible to selectively promote osteoblast functions that permit cancer cell dormancy yet resist cancer cell reawakening? Understanding the mechanisms behind osteoblast involvement in these events will lead to a better understanding of cancer cell dissemination to bone, as well as progression to metastatic disease, and will also aid in the development of therapeutic drugs to block the development of macrometastatic lesions in bone.

## Figures and Tables

**Table 1:** Sources of important factors produced by osteoblasts, osteoclasts, or bone metastatic cancer cells in the ‘vicious cycle’ of bone degradation.

Factor	Osteoblast	Osteoclast	Cancer Cells
IL-6	[50,60,189,190]	--	[50,191]
IL-8	[50,176,192,193]	[176]	[176,177,194,195,196,197]
MCP-1	[50,198,199]	--	--
VEGF	[50,130,131]	--	[50,200,201]
GRO-alpha	[50]	--	--
COX-2	[202,203,204]	--	[196,199,205,206]
TGF-beta	[129,175]	[207,208]	--
IGF-1	[175]	[19,209]	--
PTHrP	--	--	[28,84,175,210,211]
IL-11	[193]	--	[177,197,206,212]
PGE-2	[213]	--	[196,214]
Cathepsin-K	--	[173,174,215]	--
Jagged-1	--	--	[188,216]
Notch-1	[216]	[188]	--
RANK-L	[84,172]	--	--
RANK	--	[84,172]	--
Endothelin-1	[217]	--	[121,217]
DKK-1	--	--	[121,218,219,220]

Cells of the bone microenvironment produce factors in response to bone metastatic breast cancer cells. The cell sources of key factors produced are listed. The dash indicates we were unable to find evidence in the literature that the molecule was produced by the indicated cell type.
